# Research on the Coupling Mechanism of the Trade Union and Human Resource Management in China

**DOI:** 10.3389/fpsyg.2022.602145

**Published:** 2022-07-19

**Authors:** Fan Xuexiu, Sun Jiandong

**Affiliations:** ^1^School of Foreign Language and International Business, Guilin University of Aerospace Technology, Guilin, China; ^2^School of Management, Guilin University of Aerospace Technology, Guilin, China

**Keywords:** trade union, human resource management, coupling mechanism, win-win cooperation, labor relation

## Abstract

With the reform of trade unions in China, there has been a noticeable interaction in recent years between trade unions and human resource management in the workplace. However, we do not have a proper concept to describe such a phenomenon. Therefore, based on reviewing the current literature involving trade unions and human resource management in China, this study aims to describe the interactive relation between union's practices and enterprises' human resource management by exploring the definition, characteristics, and formation mechanism. This study defines the relationship between the trade unions and human resource management as a coupling relationship where the trade unions and human resource management continually promote and restrain each other as two relatively independent systems in the enterprise. This relationship possesses three dual characteristics, namely reciprocity vs. restraint, integrity vs. independence, and stability vs. dynamism. And there are three sequential stages for trade unions and human resource management to form such a relation, interaction, consensus, and adjustment. This study provides further insight into the relationship between trade unions and human resource management in the workplace and attempts a proximately complete description of the relationship. The study also offers a direction for trade unions and enterprises to establish high-quality cooperation with each other which is mutually beneficial for enterprises' development and employees' wellbeing.

## Introduction

Since the 1980's, the concept of human resource management has gained visibility. Compared to the previous type of managing employees through personnel management, human resource management (HRM) pays more attention to activating employees' potential and increasing employees' organizational commitment. By adopting HRM, numerous enterprises have successfully improved the work efficiency of employees and reduced employee turnover achieving increased profits. Furthermore, with continuous developments in HRM, western enterprises have come to realize that efficient HRM practices can effectively counter trade unions. In fact, trade unions have gradually declined under the “substitution effect” of HRM. This has not only led to a decrease in the bargaining power of the laborers in the workplace but also caused a decline in understanding of how to build an equitable and harmonious relationship between employers and employees (Kim, 2017). The lack of studies on the relationship between HRM and trade unions makes it arduous to obtain a comprehensive image of the workplace, and therefore, impossible to figure out a suitable strategy to create a better environment for both employers and employees (Guest, 2017).

In recent years, however, some researchers have argued that human resource management, like a high-performance work system, will continue to increase the workload for employees while negatively impacting their wellbeing, since enterprises always place more job demands on employees in the process of adopting human resource management practices (Han et al., 2020). Few researchers also found that when there is a trade union in the workplace, human resource management always generates better outcomes. For example, Rubinstein (2001) found that in enterprises with trade unions, human resource management can often achieve better results. Other research also found that when there is no trade union in the enterprise, the discrepancy between the enterprise and employees led to a severe imbalance between labor and capital (Kai and Brown, 2013), causing conflicts to accumulate, which eventually reduced the effectiveness of human resource management practices (Cogin et al., 2016). However, trade unions are not simple situational factors in human resource management, and there exists a complex interaction between trade unions and human resource management. For example, Cook et al. (2017) used the British retail industry as an example to explain in detail how trade unions increased the efficiency of human resource management by correcting and promoting.

In China, especially after the reform of unions under government control, the unions play two roles in the workplace: protect the interest of the employees, but also undertake the responsibility of maintaining a harmonious atmosphere thereby promoting the production of the enterprise. To make unions perform their duties better, the local union always sets up a sub-union in the enterprise, influencing the substantial interaction between HRM and the union (Snape and Chan, 2018). For example, Wang and Lien (2018) found that trade unions can effectively increase the overall wage levels of employees. Lee et al. (2016) argued that trade unions can enhance the effectiveness of human resource management by improving the atmosphere in the workplace or by relaying communication from the employees, like the demand of employees, to the human resource management department. Other studies have found that human resource management can also have an impact on the practice of trade unions. For example, Chen et al. (2018) found that since most of the trade union staff are enterprise employees, the interference of human resource management in the appointment of the members of the trade union impedes the role of trade unions, such as protecting employees' right.

This interaction between trade unions and human resource management is very similar to the coupling between systems in physics, where two systems are interdependent and interact with each other. We call this relationship between them a coupling relationship. In the following sections, we discuss the relationship between trade unions and human resource management through the coupling theory, and elaborate on this relationship with three questions: First, what is the coupling relationship between the trade unions and human resource management? Second, what characteristics does this coupling relationship possess? Third, how does this relationship emerge?

## Methods

We conducted a state-of-the-art literature review on the subject of the relationship between trade unions and human resource management, to clarify the definition of coupling between trade unions and human resource management and understand the features of the coupling. We then undertook a semi-systematic literature review (Snyder, 2019), similar to the state-of-the-art literature review approach (Grant and Booth, 2009), to explore the coupling of trade unions with HRM, and how the coupling was characterized. This method of conducting a literature review offers “the ability to map a field of research, synthesize the state of knowledge and create an agenda for further research” (Snyder, 2019, p. 335) and provides “new perspectives on an issue” (Grant and Booth, 2009, p. 105), rendering it suitable for exploratory studies like the one presented here.

First, we used the following criteria to select studies: field of study (i.e., management (including HR) and industrial relations, organization studies, and organizational behavior/psychology); source (i.e., peer-reviewed journal articles); and topic (i.e., relationship between enterprise and union, relationship between HRM and union, relationship between manager and union). A total of 44 academic studies were selected to be reviewed.

Next, we reviewed the studies and wrote down descriptions of how HRM and the unions affect each other, how HRM and the unions coexist with each other, how the relationship between them changes, etc. And then, we categorized these descriptions. We also reviewed the materials in the same category again and analyzed the mechanism of how HRM and the unions affect mutually in different aspects. Finally, the authors checked if the proposed relationship construct was able to reflect the image conveyed in the materials.

## Definition of Coupling Relationship Between Trade Unions and Human Resource Management

The concept of “coupling” comes from physics, which refers to a relationship in which two or more forms or systems of motion influence each other and even unite through interaction. After Weick (1976) first introduced the coupling theory to social science research, it has been used in many disciplines to describe the complicated relationship between systems. Coupling theory holds that two close social systems will inevitably interact with each other in three main dimensions: reductionism, holism, and evolution (Han et al., 2020). The reductionism dimension focuses on how coupled systems influence each other through interaction and defines coupling as the interaction between systems through the flow of elements. Interaction can take the form of mutual promotion or mutual inhibition (Valdivia et al., 2012). The holism dimension regards the coupled systems as a discrete whole and describes coupling as the interdependence, cooperation, or supplement between the systems (Valdivia et al., 2012). The evolution dimension views coupling as temporal, where the description of the coupling relationship should not only describe the characteristics at a specific point in time but also pay attention to the law of the coupling relationship between systems that are changing with time (Liu et al., 2005).

Similarly, in the context of Chinese enterprises, we can describe the coupling relationship between the trade unions and human resource management through the three dimensions of reductionism, holism, and evolution.

First, from the perspective of reductionism, the relationship between the trade unions and human resource management is not complete cooperation or confrontation, but a relationship within which cooperation and confrontation coexist, and the interaction between the two includes promotion and inhibition. Second, from the perspective of holism, the two parties cooperate and complement each other and operate in the overall form. In other words, HRM and the unions always work as a system, and they play different roles in the same event cooperatively. For instance, aiming to raise the performance of retail sellers of a chain store in the UK, the HRM manager makes the work process, while the union head conveys and explains the rules, and collects feedback from employees (Cook et al., 2017). Although HRM and the unions still maintain their independence from each other, they work as functional parts of the same system. Third, from the perspective of evolution, the coupling relationship between the trade unions and human resource management changes with time. Just as the activities of the trade unions and HRM are adjusted to adapt to the external environment, so also the coupling relationship between trade unions and HRM adapts in response to external policies and economic environments, like Trade Union laws, Labor laws, the market situation, and others. When the external policy and economic environment are stable, the two sides maintain a stable coupling relationship in continuous interaction. When the external environment changes, the two sides enter a new round of adaptation until a stable coupling stage is formed once again. Therefore, the coupling between the two sides is a long-term dynamic relationship interspersed with periods of stability.

To sum up, the coupling of trade unions and HRM refers to a relationship where they are continually promoting and restraining each other as two relatively independent systems in the enterprise.

## The Characteristics of the Coupling Relationship

The definition of the coupling relationship between trade unions and human resource management referred to three dual characteristics: reciprocity and antagonism, integrity and independence, and stability and dynamism. The perspective of reductionism that emphasizes the interactions between elements that coexist in the same system is an indispensable perspective to describe how the systems work. After analyzing how HRM and unions interact with each other, we concluded that they promote and restrain each other simultaneously. Therefore, we proposed that reciprocity and antagonism is the first characteristic of the coupling. From the perspective of holism, elements in a system are different parts of the same system, so the description of how elements function as one integrated system is another essential dualistic characteristic of the coupling relationship. Reviewing existing literature, we proposed that HRM and unions work cooperatively as a system in the workplace, but also maintain their own individual goals. Therefore, we concluded that being integral and independent was another dualistic characteristic of the coupling relationship. Lastly, from the perspective of evolution, although HRM and unions permanently stay in a state in which they simultaneously promote and restrain each other and are integral and independent at the same time, their specific roles and interests always change over time. So, stability and dynamism is the last dualistic characteristic of the coupling relationship.

### Reciprocity and Antagonism

Reciprocity refers to mutual promotion or support between the trade unions and human resource management. Trade unions promote HRM in three ways: first, trade unions, working directly with the employees, represent the reasonable demands of employees to the management. Based on the representation of demands by trade unions, the enterprise can adjust and optimize its human resource management (Cook et al., 2017). Secondly, trade unions serve as a channel of communication beyond the HRM in helping employees communicate their problems at work to the senior management of the enterprise. Situations where supervisors ridicule, abuse, or snub employees are thus effectively reduced, ensuring the smooth operation of human resource management (Cook et al., 2017). Thirdly, by organizing recreational activities such as friendly competitions, painting contests, or providing personal care for employees, like celebrating employees' birthdays, the trade unions can create a relaxed and harmonious atmosphere in the workplace, improving employees' impression of the enterprise, and making HRM more acceptable to employees (Chen et al., 2018).

Similarly, HRM supports trade unions to function smoothly by providing the necessary resources for them to carry out their work. First, a series of training workshops on educational attributes, like skill competition, hosted by trade unions not only enhance the work skills of employees but also improve the working atmosphere in the enterprise (Chung, 2016). Therefore, HRM usually supports the implementation of these activities, such as providing the necessary venue and funds and expects such activities to improve the efficiency of the employees (Chan et al., 2017). Secondly, the HR department of the enterprise, which has professional knowledge and skills, can provide guidance and suggestions for the trade union to establish the employee collective bargaining system, and enhance the effectiveness and rationality of the collective bargaining mechanism in the enterprise (Taylor and Li, 2010).

At times, trade unions and human resource management also restrict each other thereby indicating elements of antagonism in their relationship. In the process of production and operation, enterprises usually reduce labor costs and increase the efficiency of the enterprise through some HRM practices like increasing work intensity, extending working hours, and reducing wages. To avoid the infringement of employees' rights and interests, trade unions often help to raise the wages of employees, reduce their working hours, and strive for a better working environment through collective contracts, collective voice, and collective bargaining (Budd et al., 2014). This forces HRM to pursue the efficiency of the enterprise while at the same time ensuring employees' rights and interests, despite reducing the degree of freedom of human resource management.

Likewise, at times, HRM also restricts the functioning of trade unions. Chinese trade unions are embedded within enterprises and exist in the form of a department. Trade union staff often have administrative positions in enterprises, and their salaries, rewards, and job transfers are all controlled by human resource management (Chang and Cooke, 2015). Therefore, when trade unions represent employees to defend their rights, they also need to take into account that it does not affect the production efficiency and therefore use soft skills such as negotiations and suggestions (Lee et al., 2016).

### Integral and Independent

Coordination and cooperation ensuing between the trade unions and human resource management demonstrate the integral element in their relationship. First, the main components of HRM include salary, reward, training, selection, and performance management (Guest, 2017), focusing on the hierarchical (vertical) arrangement of the employees' work life. And for the trade unions, apart from protecting employees' rights, their duty also includes caring for employees' lives and carrying out recreational activities, focusing on serving the employees, and improving their experience in the workplace (Hu et al., 2018) which are all aimed at the employees' horizontal interactions at the workspace. Therefore, the excellent complementing cooperation between unions and human resource management in Chinese enterprises is reflected in the way HRM makes reasonable arrangements for employees at work, and the trade union focuses more on caring for the employees, carrying out welfare activities, and protecting employees' right to know (Yao and Zhong, 2013). Secondly, because the activities of the trade unions are directly oriented toward employees and they have inside knowledge of the employees' needs, enterprises often cooperate with trade unions in job design, job training, and recruitment management, in order to take into account the needs of both employers and employees as a whole and make strategies in line with the current business conditions of enterprises (Friedman, 2012).

However, at the same time, trade unions and human resource management also maintain their independent service objectives and goals in the process of adjusting to each other. While trade unions focus on protecting the rights of employees, human resource management pays more attention to ensuring there is synergy between the rights and interests of employees and their work efficiency. This is done by stimulating employees' work enthusiasm and increasing employees' work efficiency by increasing employees' organizational commitment, sense of responsibility, and similar other ways instead of using methods like overtime work, wage reduction, etc. (Wood, 1996). However, human resource management substantially serves the enterprise, and its primary purpose is to improve the efficiency of the enterprise (Collings et al., 2018).

As for trade unions in Chinese enterprises, because the salaries of union cadres and union's funding sources are controlled by HRM, they do not engage in disruptive ways of protecting employees' rights such as strikes and parades that affect the productivity and operation of enterprises, and instead, use softer approaches such as collective bargaining, signing collective contracts, and collective voice to fight for the rights and interests of employees (Lee et al., 2016). Besides, the “maintenance of order” function of the enterprise trade unions, which is stipulated in the Trade Union Law, also requires it to assume the responsibility of maintaining the stability of enterprise production and mobilizing productivity in employees (Wang and Zheng, 2012). However, at present, trade unions still regard serving employees and protecting their rights and interests as their primary responsibility (Qi et al., 2021). This demonstrates that through the process of interaction between the trade unions and human resource management, each other's content and method of engagement have changed, but their purpose and service objective have not changed. Trade unions and human resource management remain relatively independent.

### Stability and Dynamism

Through continuous influence, trade unions and human resource management eventually settle into a stable phase of coupling. However, when the external environment changes, they adjust and adapt responding to the changes in the external environment until they arrive at a new stable phase of coupling. For example, before 1990, the distribution of interests between enterprises and employees in Chinese state-owned enterprises was directly determined by the government, and there were no conflicts caused by interest distribution in enterprises. Trade unions and human resource management cooperated in carrying out their activities to increase the overall benefit of the enterprise. The trade unions not only cooperated with the enterprise in training and recruitment but also actively assumed the responsibility of mobilizing staff production. At that point, the trade unions and HRM showed a particular model of a stable phase of coupling (Chang, 2013). However, this stable phase has changed with the changes in external policies and markets. With the reform of state-owned enterprises after 1992, enterprises became responsible for their profits and losses and gradually put the interests of themselves and investors in the first place, resulting in frequent refusals or violations of labor contracts, low wages, poor working conditions, and so on, which dramatically infringed upon the rights and interests of the employees. Responding to this situation, trade unions adjusted their focus and began to make concerted efforts to safeguard employees' rights. On the other hand, enterprises attempted to restrict the trade unions' agenda of safeguarding the rights of employees by putting pressure on the unions or interfering with the employment of trade union workers (Zhu et al., 2011). This triggered a new round of mutual adjustment and adaptation until the two sides formed another stable coupling relationship.

## Establishment of the Coupling Relationship

With the objectives of trade unions and human resource management being different, their respective focus remains different. However, both sides equally uphold the purpose of increasing enterprise efficiency and ensuring employee welfare at the same time. So a win-win between labor and capital is the ultimate goal that is pursued by both sides. This goal drives the mutual promotion and cooperation between trade unions and human resource management. But the difference in their respective focus urges each side to continually regulate and restrain the activities of the other side. When the external environment is stable, the interaction between the two sides ensures that a balance is maintained; when the external environment changes, the two sides adjust through continuous interaction to ensure the stability of labor relations in the enterprise.

### Consensus

Human resource management, which serves the enterprise, including a series of practical activities formulated and carried out for the profit of the enterprise, emphasizes improving the work efficiency of employees through the control and motivation of employees (Blom et al., 2019). Therefore, the task of human resource management usually includes job design, staff recruitment, performance appraisal, and salary management. While these help in standardizing employees' behavior and increase their work efficiency, it eventually helps enterprises to increase their productivity (Sun et al., 2007). At the same time, HRM also ensures the participation, authorization, and job security of employees, to ensure fairness in the workplace, maintain stable labor relations and prevent turnover, strikes, and other events that affect the productive efficiency of enterprises (Jensen et al., 2011). Thus it can be seen that although the ultimate goal of human resource management is to improve enterprise performance, ensuring the fairness of employees to a certain extent is an equally important goal.

For trade unions, protecting the rights and interests of the employees has always been its fundamental duty. Moreover, after the promulgation of the three Labor laws in 2010 in China (namely, Labor Contract Law, Employment Promotion Law, Labor Dispute Mediation and Arbitration Law), serving employees and ensuring employee fairness has become the main work of trade unions in this stage (Cooke, 2014). However, Article 7 of the Trade Union Law also stipulates that “trade unions mobilize and organize workers to participate in economic construction actively and strive to complete production tasks and work tasks.” Article 29 of the Trade Union Law also stipulates that “when there is a stoppage or idling event in an enterprise or institution, the trade union shall communicate with the enterprise, institution or relevant parties on behalf of the staff and workers, reflect the opinions and requirements of the workers and put forward solutions. Enterprises and institutions shall address the reasonable requirements of the staff and workers. Trade unions assist enterprises and institutions to restore production and work order as soon as possible.” Therefore, helping enterprises maintain production order and improve production efficiency is also one of the responsibilities that trade unions need to undertake at present.

As observed, although the differences in service objectives lead to different focus of work between trade unions and human resource management, both sides are committed to the dual goals of increasing enterprise performance and ensuring employee fairness. A win-win between labor and capital is the ultimate goal of both parties.

### Interaction

The win-win goal of labor and capital promotes mutual support and cooperation between the two sides, while the difference in their respective focus serves to regulate the practice of the other side through mutual restraint.

The goal of a win-win between labor and capital drives trade unions and human resource management to increase their respective functions through mutual support and cooperation. Trade unions enhance the effect of human resource management practice by conveying employees' demands and supervising grassroots managers, which can not only improve the efficiency of enterprises but also protects the rights and interests of the employees. Human resource management is also expected to provide support for recreational activities, skills competitions, and other trade union activities, hoping to enhance the enthusiasm among employees, and improve enterprise efficiency, which also improves the workplace experience of employees. Also, the goal of a win-win between labor and capital has further promoted the division and cooperation between trade unions and human resource management. For example, in enterprises, human resource management is usually used to make reasonable work arrangements for employees, while trade unions undertake the task of addressing employees' emotions, protecting employees' right to know, and conveying employees' demands. At the same time, fixing wages, training, and other works are also carried out by trade unions and human resource departments to coordinate the needs of both employers and employees, to ensure the sufficient coordinated growth of the interests of both sides.

On the other hand, the difference in the focus of the goal urges the two sides to regulate each other. The responsibilities of the unions require them to protect the rights and interests of employees through collective agreement, collective voice, and collective bargaining and ensure that employees can be treated fairly by the enterprise, including reasonable salary, working hours, and a healthy working environment. In this way, trade unions limit the scope and manner in which HRM can function in the enterprise. At the same time, human resource management also interferes with the employment of trade union personnel to ensure that the trade unions' activities are carried out without affecting the productive efficiency of the enterprise. Trade unions and human resource management thus continuously undergo adjustments in response to the restrictions placed by each other and resume their activities while respecting each other's goals. That is, human resource management of the enterprise pursues efficiency without harming the rights and interests of employees; meanwhile, the rights protection practice of trade unions uses softer approaches like suggestions and negotiations, to ensure that the production and operation activities of enterprises are not affected.

### Adjustment

When the external economic and policy environment changes, such as the revision of the Labor Law, the Trade Union Law, or the change of the external industry environment, the existing human resource management or the operation mode of the trade unions can no longer guarantee the achievement of the objectives of the system or cannot meet the survival needs of the main body served by the system. The enterprise or trade unions will take the initiative to adjust human resource management or unions to adapt to the changes in the environment and cause the other party to respond and make corresponding adjustments to ensure the stability of labor relations in the enterprise.

For example, in 2015, when the coal market remained in the doldrums, the Furong Company of Sichuan Coal Group encountered discontinuous production and continuous losses. To ensure the continuity of the enterprise's capital flow, Furong reduced the wages of its employees and adopted a policy of delaying the payment, which led to increasing tensions in what was once a harmonious labor relationship. After understanding the predicament of the enterprise in its process of negotiation with them, the trade union did not forcibly use the legal route or resort to the superior strength of the trade. Instead, it actively negotiated with the enterprise and determined the work goal of shared destiny, and overcame the difficulties together. The enterprise agreed to pay part of the salary temporarily to solve the difficulties of the employees. At the same time, the trade union actively helped the enterprise to share its difficulties with the employees, appeased the employees' emotions, and took the initiative to use trade union funds to address the employees' life and medical difficulties. Simultaneously, the enterprise and trade union cooperated to promote employees' participation in the enterprise's management, increase employees' sense of belonging, and actively solicited suggestions from employees to plug loopholes in the enterprise's production in time to reduce production costs. Due to the timely recalibration of work objectives and the active cooperation of the trade union and human resource management of the enterprise to take reasonable measures, Furong Company finally got out of the production predicament and stabilized its production and operation.

## Discussion

### Theoretical Implication

There are two main theoretical contributions made by this study. First, this study has provided a more succinct description of the relationship between trade unions and human resource management. Previous research always regarded trade unions and human resource management as two independent parties and discussed the relationship between them from the perspective of comparing each other's strength. And arguing that only if there existed a balanced strength between trade unions and enterprise, there could be conducive cooperation between them (Hernaus et al., 2019). However, such a static description merely shows an approximate image and neglects the deeply layered interaction between the trade unions and human resource management. The results of this study describe the coupling relationship between trade unions and human resource management. It also offers the definition, characteristics, and formation process of the relationship, and details the exhaustive description of such a relationship. It provides an alternative perspective to explore the nature of the relationship between trade unions and human resource management.

Second, this study enriches the domain of conjunction of industrial relations and human resource management. Researchers who hold monistic beliefs argue that human resource management can solve all the employer-employee problems in the workplace since they believe there is no inevitable conflict between employer and employee (Guest, 2017). However, recent research indicates that human resource management is detrimental to employees' work-family balance, wellbeing, and health (Boxall and Macky, 2014). This study echoes the call for pluralism, integrating trade unions into the framework of enterprise management to reconcile the potential negative impact of human resource management on employees, giving a framework to understand how these two systems interact with each other, enriching the domain of how to use pluralism to construct a win-win management system.

### Practical Implication

First of all, enterprises should respect the independence of trade unions and regard their work, like protecting the rights of employees, as vanguards of their own HRM. The human resource management of the enterprise is usually formulated and implemented from top to bottom, which causes it to pay more attention to the interests of the enterprises and often ignore the rights and interests of employees, which can easily lead to turnover, strikes, and other issues. Moreover, the process of trade unions helping employees to protect their rights is also a process of conveying employee demands to enterprises, thereby providing an opportunity for the enterprises to adjust and optimize their HRM based on communication received from the trade unions. When the enterprise completely suppresses trade unions and makes them a vassal of the HRM, the trade unions cannot protect the rights of employees and lose their trust, and consequently, cannot help the enterprise to obtain the reasonable demands of the employees. Therefore, enterprises should support the activities of trade unions and carefully respond to the advice from employees communicated through their unions. Helping the trade unions to hold the employee congress is another effective measure to build the trust toward enterprise in the heart of the employees.

Secondly, trade unions should actively help employees fight for their rights and interests above the bare legal requirement, instead of relying on the bottom line to protect their rights passively. Currently, the Chinese government has made explicit provisions for the rights and interests of employees at the employment site, which, to a certain extent, gives trade unions not only legal support but also a reference standard to protect the rights of employees. However, such standards are formulated by the government according to the average level of weak companies, with a particular nature of relief, and cannot adequately meet the needs of all employees. Especially in recent years, workers under 40 years of age have become the leading force in the workplace. The awareness of safeguarding the rights of employees is gradually rising, and they have begun to pursue more career growth rights and interests. Relying on the legal bottom line is just negative protection of rights that may be regarded as the union's neglect of employees' rights and interests, resulting in labor disputes. Only when trade unions engage deeply with the employee groups, understand their demands, and help them strive for reasonable growth rights and interests from the enterprise, can the trade unions help the enterprise build harmonious labor relations.

Finally, for both trade unions and enterprises, when they need to adjust their respective activities due to changes in external policies and the economic environment, they should actively communicate with each other instead of unilaterally changing their activities. Not doing so would destroy the existing stable coupling relation between them and trigger unnecessary negative consequences. Only mutual respect and good communication can ensure that the unions and human resource management maintain a stable and benign coupling relationship. When the external environment changes, they need to maintain a balance between efficiency and fairness within the enterprise to ensure the long-term and stable development of the enterprise.

## Limitations and Future Research

This study is not without limitations. First, there is a lack of empirical analysis. This study proposes a coupling relationship between the trade unions and human resource management, defines the relationship, describes the features of this relationship, and analyzes the formative process. However, empirical research is an important underpinning to validate the relationship proposed by this study. Second, a more universal discussion between trade unions and human resource management is required. This study is based on research in the context of Chinese trade unions and human resource management in Chinese enterprises. Although the results of this study can offer a reference for future researchers to explore the relationship between trade unions and human resource management, the value of this study may be limited by the context of the discussion.

Therefore, there are some suggestions for future research stemming from the limitations of this study. First, future research can use content analysis to certify the proposition of this study, including the characteristics of the coupling relationship between trade unions and human resource management and the formative phase of such a relationship. Second, future research can employ cases or samples from western countries or other eastern countries, like Japan and Korea, to further explore the relationship between trade unions and human resource management, and establish a more robust framework to describe the relationship.

## Data Availability Statement

The original contributions presented in the study are included in the article/supplementary material, further inquiries can be directed to the corresponding author.

## Author Contributions

SJ contributed to the idea, writing, and revision of this article. FX contributed to the revision of the article. All authors contributed to the article and approved the submitted version.

## Funding

This study was supported by the University-Level Scientific Research Platform of Guilin University of Aerospace Technology: Research Center for Digital Economy Development in Underdeveloped Areas.

## Conflict of Interest

The authors declare that the research was conducted in the absence of any commercial or financial relationships that could be construed as a potential conflict of interest.

## Publisher's Note

All claims expressed in this article are solely those of the authors and do not necessarily represent those of their affiliated organizations, or those of the publisher, the editors and the reviewers. Any product that may be evaluated in this article, or claim that may be made by its manufacturer, is not guaranteed or endorsed by the publisher.
