# Corrigendum: Research on the coupling mechanism of the trade union and human resource management in China

**DOI:** 10.3389/fpsyg.2025.1622298

**Published:** 2025-05-28

**Authors:** Fan Xuexiu, Sun Jiandong

**Affiliations:** ^1^College of Economics and Management, Nanjing University of Aeronautics and Astronautics, Nanjing, China; ^2^School of Foreign Language and International Business, Guilin University of Aerospace Technology, Guilin, China; ^3^School of Management, Guilin University of Aerospace Technology, Guilin, China

**Keywords:** trade union, human resource management, coupling mechanism, win-win cooperation, labor relation

In the published article, there was an error regarding the affiliations for Fan Xuexiu. As well as having affiliation 2, “School of Foreign Language and International Business, Guilin University of Aerospace Technology, Guilin, China,” they should also have the new affiliation 1, “College of Economics and Management, Nanjing University of Aeronautics and Astronautics, Nanjing, China.”

The authors apologize for this error and state that this does not change the scientific conclusions of the article in any way. The original article has been updated.

